# A Cross-Sectional Analysis of the Association between Domestic Cooking Energy Source Type and Respiratory Infections among Children Aged under Five Years: Evidence from Demographic and Household Surveys in 37 Low-Middle Income Countries

**DOI:** 10.3390/ijerph18168516

**Published:** 2021-08-12

**Authors:** Zubaidah Al-Janabi, Katherine E. Woolley, G. Neil Thomas, Suzanne E. Bartington

**Affiliations:** Institute of Applied Health Research, University of Birmingham, Edgbaston, Birmingham B15 2TT, UK; ZXA945@alumni.bham.ac.uk (Z.A.-J.); KEW863@student.bham.ac.uk (K.E.W.); s.bartington@bham.ac.uk (S.E.B.)

**Keywords:** biomass cooking, household air pollution, acute respiratory infection, low and middle-income countries

## Abstract

Background: In low- and middle-income countries (LMICs), household air pollution as a result of using solid biomass for cooking, lighting and heating (HAP) is associated with respiratory infections, accounting for approximately 4 million early deaths each year worldwide. The majority of deaths are among children under five years. This population-based cross-sectional study investigates the association between solid biomass usage and risk of acute respiratory infections (ARI) and acute lower respiratory infections (ALRI) in 37 LMICs within Africa, Americas, Southeast Asia, European, Eastern Mediterranean and Western Pacific regions. Materials and methods: Using population-based data obtained from Demographic and Health surveys (2010–2018), domestic cooking energy sources were classified solid biomass (wood, charcoal/dung, agricultural crop) and cleaner energy sources (e.g., Liquid Petroleum Gas (LPG), electricity, biogas and natural gas). Composite measures of ARI (shortness of breath, cough) and ALRI (shortness of breath, cough and fever) were composed using maternally reported respiratory symptoms over the two-week period prior to the interview. Multivariable logistic regression was used to identify the association between biomass fuel usage with ARI and ALRI, accounting for relevant individual, household and situational confounders, including stratification by context (urban/rural). Results: After adjustment, in the pooled analysis, children residing in solid biomass cooking households had an observed increased adjusted odds ratio of ARI (AOR: 1.17; 95% CI: 1.09–1.25) and ALRI (AOR: 1.16; 95% CI 1.07–1.25) compared to cleaner energy sources. In stratified analyses, a comparable association was observed in urban areas (ARI: 1.16 [1.06–1.28]; ALRI: 1.14 [1.02–1.27]), but only significant for ARI among those living in rural areas (ARI: 1.14 [1.03–1.26]). Conclusion: Switching domestic cooking energy sources from solid biomass to cleaner alternatives would achieve a respiratory health benefit in children under five years worldwide. High quality mixed-methods research is required to improve acceptability and sustained uptake of clean cooking energy source interventions in LMIC settings.

## 1. Introduction

In low- and middle-income countries (LMICs), household air pollution (HAP) is associated with acute respiratory infections (ARI) and acute lower respiratory infection (ALRI) as a result of using solid biomass for cooking, lighting and heating [1,2]. Solid biomass fuels, including coal, charcoal, crop waste and dung, are used by more than three billion people worldwide primarily in LMIC settings due to widespread availability and lower cost [3]. However, these fuels produce harmful levels of pollutants, including particulate matter (PM), carbon monoxide (CO), sulphur dioxide (SO_2_) and nitrogen oxides (NO_x_). Inequalities in the level of exposure are present between different household members, with women and children experiencing the highest exposure levels [4], due to women performing the majority of domestic duties including the preparing and cooking of food [5] typically accompanied by young children during cooking periods [6,7]. This high exposure in children under five years increases their risk of ARI [8], asthmas [9], stunting [10], otitis media [11], poor neurodevelopment [12] and mortality [10], with over four million deaths associated with HAP exposure each year [13].

Interventions to reduce HAP exposure, including Liquefied Petroleum Gas (LPG), Biogas, Improved cookstove (ICS), biomass pellets and behaviour change (e.g., removing children from the cooking area) [14], have indicated some health-related benefits throughout the life-course in some countries [15]. Implementation of such interventions faces multiple challenges in LMIC settings, due to lack of financial resources, awareness and accessibility [15,16]. Imposing changes to adopt cleaner cooking energy sources is challenging, with high start-up costs and need for ongoing investment in changing household behaviours in LMICs [17]. However, HAP interventions present a number of longer-term benefits including economic savings, despite the upfront costs [17]; environmental benefits such as a reduction in deforestation [18]; reduced opportunity costs [19]; and wider social benefits through gender equality, female empowerment and additional time for education [19].

Previous research identified a strong association between the use solid biomass cooking fuel and reduced risk of respiratory infections in India [20,21], Pakistan [22] and Africa [23], especially among households with less educated parents [24]. However, to the best of our knowledge, there is a paucity of evidence of ARI risk between solid biomass and cleaner cooking energy sources on a global scale taking into consideration both ARI and ALRI. Utilising data obtained from Demographic and Health Survey (DHS) for 37 LMICs worldwide, this paper aims to investigate differences in the association between solid biomass and cleaner cooking energy sources with risk of ARI and ALRI among children aged under five years old.

## 2. Materials and Methods

### 2.1. Setting and Study Design

Data were obtained from the nationally representative population-based demographic and health survey, funded by U.S. Agency for International Development and other participating countries [16], collected repeated survey every four years from over 90 countries. Each survey has a multi-stage stratified sampling strategy that provides high-quality information on family planning, fertility, maternal and child health, nutrition and other living condition aspects [25].

**Figure 1:**
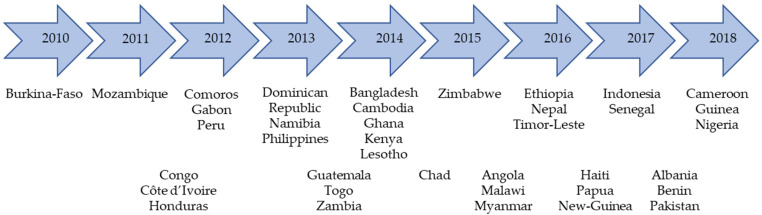
Countries and DHS questionnaire dates included in this study.

For this cross-sectional study, relevant data for children under five years old were extracted from the most recent DHS surveys conducted between 2010 to 2018 for countries with available data resulting in the selection of 37 completed surveys (Figure 1).

### 2.2. Ethical Approval and Authorisation

ICF Institutional Review Board (IRB) and individual country government ethical approval board, provided ethical approval for survey data collection. All data are anonymised and made publicly available on the DHS website [26]. Data access authorisation was provided by DHS [27].

### 2.3. Data Variables

#### 2.3.1. Outcome/Dependent Variables

ARI and ALRI are composite outcome measures derived from maternal report of respiratory symptoms (cough, short rapid breath or difficulty breathing, and fever) in children under five years occurring in the two-week period prior to the survey contact. ARI was defined as the presence of cough and short rapid breaths [28], while ALRI defined as the presence when cough, short rapid breath and fever [28]; each were modelled as a binary (yes, no) outcome variable.

#### 2.3.2. Exposure/Independent Variables

Household cooking energy sources were categorised into cleaner (electricity, LPG, natural gas, biogas) and biomass (kerosene, coal/lignite, charcoal, wood, straw/shrubs/grass, agricultural crop, animal dung) fuel types [29].

#### 2.3.3. Child, Maternal and Paternal Characteristics

Children’s characteristics comprised (a) child age (<1, 1, 2, 3, 4 years old), (b) child sex (female, male), (c) mode of delivery (vaginal, caesarean) and (d) breastfeeding status (ever breastfed, never breastfed). Maternal characteristics comprised (a) maternal age (15–24, 25–35, 36–49 in years), (b) highest educational level (none, primary, secondary/higher) and (c) highest education level of husband (none, primary, secondary/higher).

#### 2.3.4. Household and Contextual Characteristics

Household characteristics considered in the analysis were (a) indoor household smoking (yes, no), (b) number of household members as a proxy for household crowding (≤6, >6), (c) household cooking location (inside, outdoors) and (d) wealth index (lowest, low, middle, high, highest). The wealth index measure used according to DHS socio-economic five categories, using wealth indicator variables collected within the household survey. Every collected household indicator variable gets assigned a factor score through principal component analysis and the resulting scores are then standardised in a normal distribution using a mean of zero and standard deviation of one. These standardised scores are then used to generate and define the wealth index categories as lowest, low, middle, high and highest [30]. Contextual characteristics included (a) rural or urban residence (urban, rural) and (b) region of residence.

### 2.4. Data Analysis

R studio [31] was used for data management, manipulation and analysis. A summary of descriptive statistics was derived using number of cases (n) and percentages (%) as all our variables were either categorical or binary. Multivariable logistic regression analysis was conducted using univariate stepwise selection with the survey package in R studio and with odds ratios (OR), 95% confidence interval (95% CI) and p values reported. Confounding factors included in the final adjusted model included child sex and age, mode of delivery, maternal age and level of education, wealth index, cooking location, number of household members, region and urban or rural residence. As some countries had low cell counts or entirely missing values for breastfeeding status, household smoking status and husband’s level of education, exploratory analysis for these factors was undertaken only among those countries where these variables were available. An additional sub-analysis stratified by rural and urban residence was also undertaken. Multicollinearity was checked by the variance inflation factor (VIF), using Car package in R studio [32].

## 3. Results

### 3.1. Descriptive Analysis

**Figure 2:**
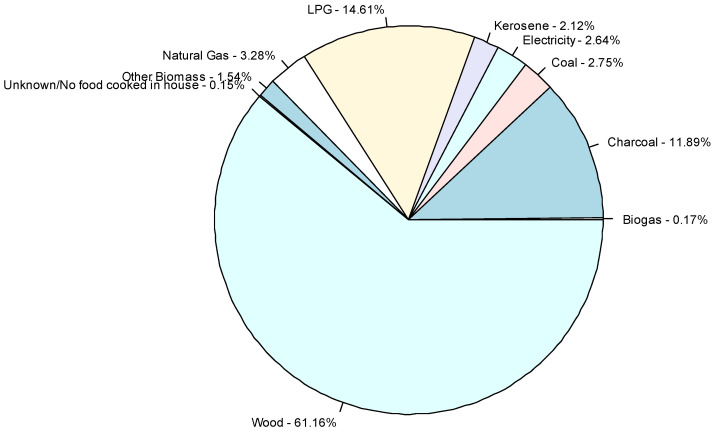
Proportion of children residing in households using different cooking energy source types within the pooled dataset.

**Figure 3:**
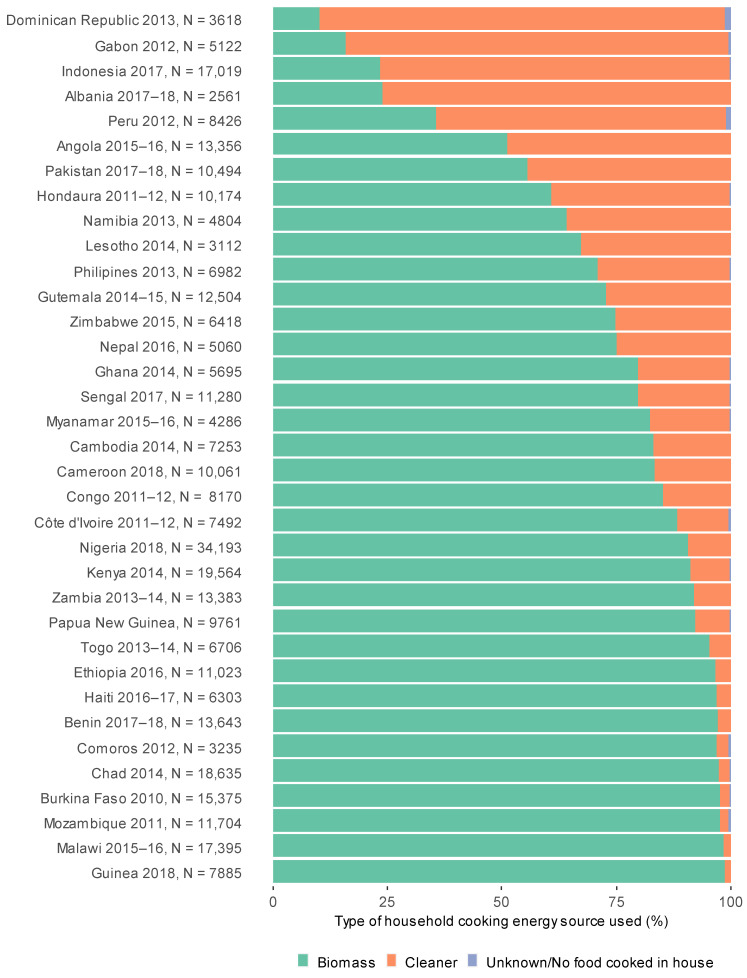
Proportion of children residing in households using cleaner and biomass cooking energy sources by country.

**Figure 4:**
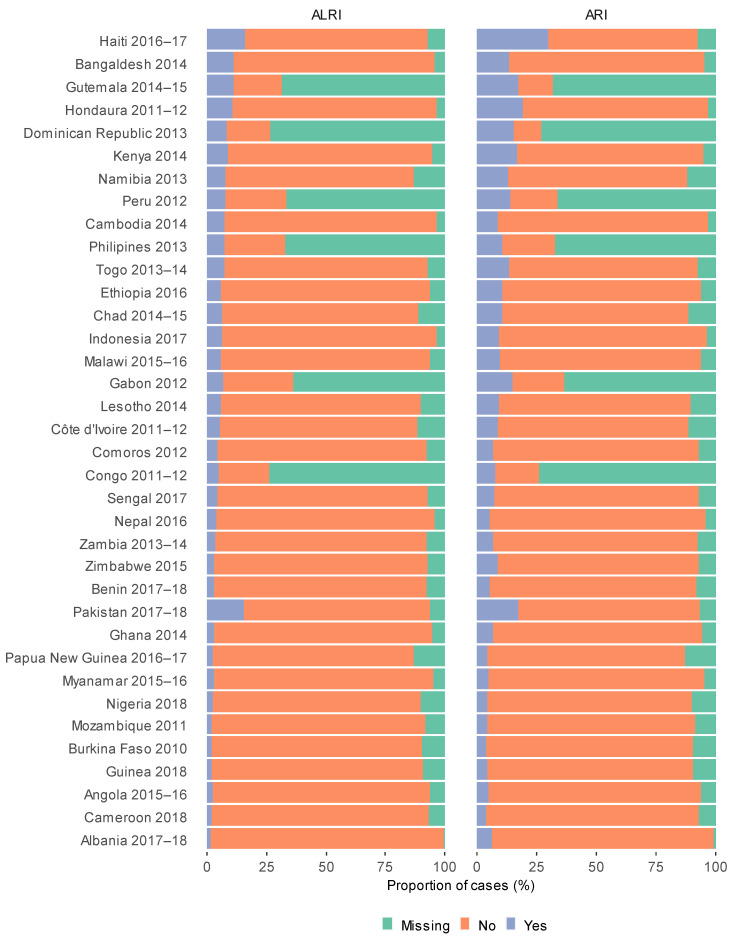
Proportion of ARI and ALRI cases in children under five by country.

In the pooled dataset of 353,802 children living in 37 countries, 79.6% lived in solid fuel cooking households and 20.4% resided in cleaner cooking energy source households (Figure 2 and Figure 3). Overall, there were 32,438 (10.8%) cases of ARI and 19,426 (6.5%) cases of ALRI occurring in the two weeks prior to interview (Table 1). Proportions of clean cooking energy sources use varied across the included countries with 88.5% using cleaner cooking energy sources in Dominican Republic compared to 1.4% in Guinea (Figure 2). Haiti had the highest period prevalence of ALRI (15.5%) and ARI (28.9%), compared to the lowest in Albania (ARI: 1.7% and ALRI: 5.5%; Figure 4).

### 3.2. Risk of ARI and ALRI in Children under Five

**Figure 5:**
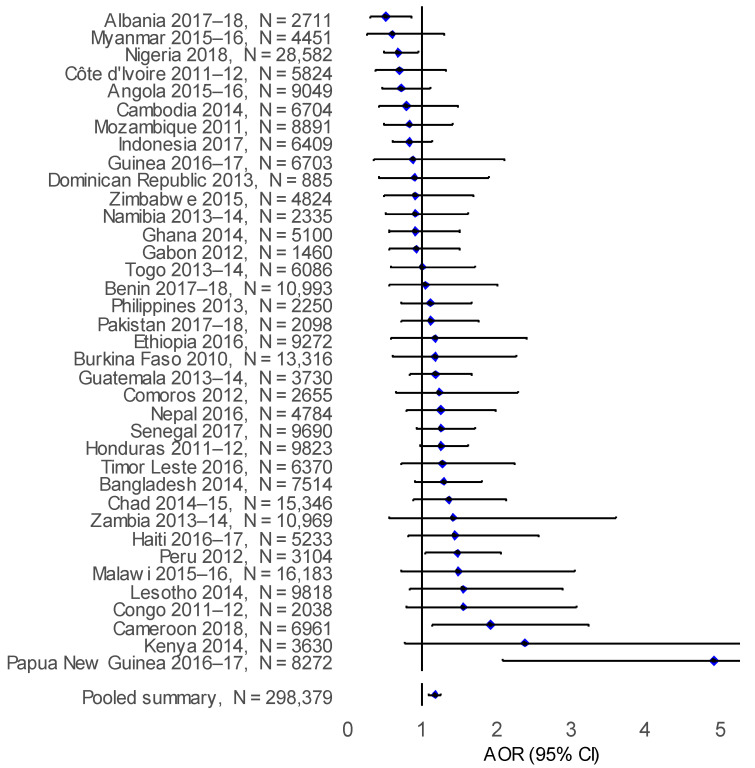
Forest plot for the adjusted odds ratio (AOR) for ARI in children aged under five years with biomass cooking compared to cleaner cooking for all countries.

**Figure 6:**
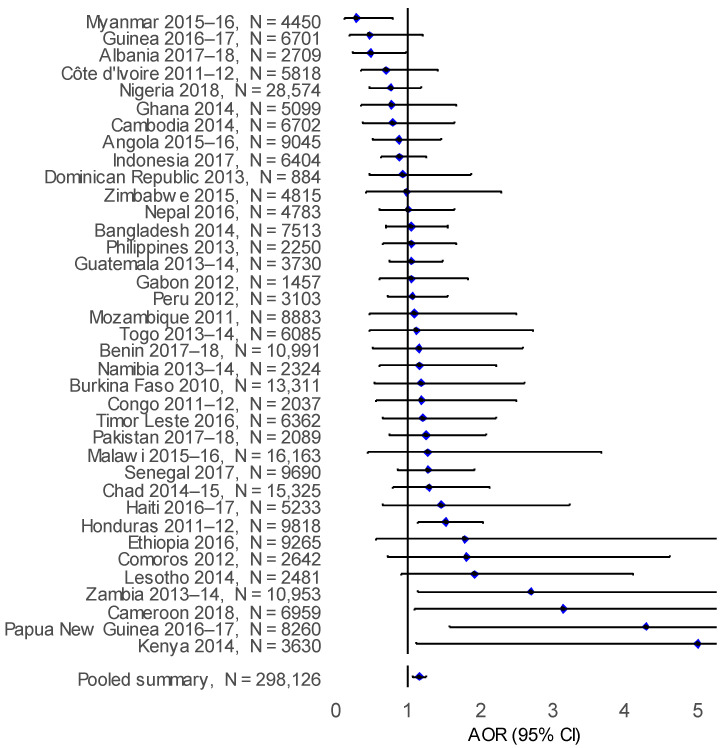
Forest plot for the adjusted odds ratio (AOR) for ALRI in children aged under five years with biomass cooking compared to cleaner cooking for all countries.

After adjusting for individual and household confounding factors, solid biomass cooking was observed to independently increase the adjusted odds ratio of ARI compared to cleaner cooking energy sources in Papua New Guinea (AOR: 4.91; 95% CI: 2.08–11.50), Cameroon (AOR: 1.92; 95% CI: 1.14–3.23) and Peru (AOR:1.47; 95% CI: 1.05–2.05) (Figure 5). In the pooled analysis, there was an observed increase the adjusted odds ratio of ARI (AOR: 1.17; 95% CI: 1.09–1.25) with solid biomass cooking compared to cleaner cooking energy sources (Table 2). However, for ALRI an increase in the adjusted odds ratio with biomass cooking was observed in Kenya (AOR: 5.00; 95% CI: 1.10–22.60), Papua New Guinea (AOR: 4.28; 95% CI: 1.58–11.60), Cameroon (AOR: 3.15; 95% CI: 1.09–9.05), Zambia (AOR: 2.69; 95% CI: 1.13–6.39) and Honduras (AOR: 1.52; 95% CI: 1.13–2.04), compared to cleaner cooking energy sources (Figure 6). A similar adjusted odds ratio was observed in the pooled analysis, with a greater risk of ALRI (AOR: 1.16; 95% CI: 1.07–1.25) associated with living in biomass cooking compared to cleaner cooking energy source households.

Within the stratified sub-analysis, similar associations were observed (Table 3). Among children living in urban areas only there was an increased risk of ARI (AOR: 1.16; 95% CI: 1.06–1.28) and ALRI (AOR: 1.14; 95% CI: 1.02–1.27) in biomass cooking compared to cleaner cooking households. An association was observed for ARI (AOR: 1.14; 95%CI: 1.03–1.26) in rural areas only, but not for ALRI (AOR: 1.13; 95% CI: 1.00–1.27) (Table 2).

## 4. Discussion

Solid biomass domestic fuels are used as a main source of energy in many countries worldwide, primarily due to the lack of access to clean energy alternatives. Our large-scale (N = 353,802) cross-sectional study conducted across 37 LMICs between 2010 and 2018 indicates that cooking with cleaner energy sources could reduce ARI and ALRI by 17% and 16%, respectively, among children under five years, compared to traditional solid biomass cooking energy source usage. We observe the benefits of clean cooking energy source alternatives to be even more pronounced among urban LMIC subpopulations, with important implications for targeted cooking energy source transition policies.

Although there is a clear benefit of cleaner cooking of improving childhood respiratory outcomes, there exists a lack of national and international policy attention to facilitating sustained access and uptake of cleaner cooking energy sources [33]. Not only have we identified substantial between-country variation in ARI risk associated with biomass cooking, but there is also widespread variation in provision of clean cooking energy source alternatives and cooking practices. In our analyses, we include household wealth to adjust for the influence of socio-economic factors operating at a household level; however, it is recognised that the macroeconomic context will influence cooking energy source accessibility and choice based upon market prices [34,35]. In addition, our analysis explored impacts of health and behavioural factors (e.g., household smoking, breastfeeding and husband’s level of education) within countries with relevant available data, identifying that these potential confounders have little influence on reducing ARI risk (13%, 14% and 13%, respectively) in children under five (Appendix A: Table A2). The complex relationship between cooking energy source choice and external situational and contextual factors is well recognised (such as traditional practices, local economic situation and cooking energy source availability) [36] and any individual country level policy should attempt to identify and capitalise upon relevant situational factors which could affect sustained clean cooking energy source uptake in the longer term.

Notably, within our study are the observed differences in the risk of ARI between rural and urban areas, with marginally higher risk in children residing urban areas, which is a well-recognised phenomenon in other settings [2], due to different situational (e.g., source of pollutant emissions, healthcare access and nutritional status) [37], and behavioural characteristics (e.g., cooking location and time spent indoors) [38]. As most children worldwide spend most of their time in indoor settings [39,40], particularly within this age group, given children under five years are typically not yet attending school, we did not attempt to capture any measures of outdoor (ambient) pollution such as household proximity to roads, industrial sites and neighbouring households cooking energy source, which may mitigate the benefits of cleaner cooking energy source use [41,42]. However, it is well recognised that the relationship between indoor and outdoor air quality is complex, influenced by factors such as housing characteristics, housing density, cooking location and ventilation mechanisms dominate emissions sources and quantities of exposure [43]. Nevertheless, as children are known to typically spend a substantial proportion of time in close proximity to the cooking location in these contexts [39,40]; therefore, this is likely to present as the major emissions source and dominant exposure microenvironment in this age group.

Clean and biomass cooking have been well defined by the WHO, however, there is internal variation within these categories, with studies indicating that there are potential respiratory health benefits associated with each step up the fuel ladder [2,44], for example, by substitution of solid biomass fuels with kerosene. The WHO previously categorised kerosene as a cleaner energy source, even though it is more polluting than LPG; some analyses have previously used this categorisation [45], limiting potential comparability. However, when exploring categorising kerosene as a cleaner energy source (Appendix A: Table A3), within the combined dataset, there was little observable effect on the over ARI risk (ARI: 20%, ALRI 19%), which could be due to the lower number of kerosene users globally. Cleaner energy source use remains infrequent in many countries, limiting the inclusion of some countries within the analysis and these countries should encourage policies to improve cleaner energy source usage. Despite this limitation 37 LMICs and 353,802 children were included in our analyses supporting published country or regional level literature on the potential respiratory health benefit of cleaner cooking energy sources [23,46,47,48,49].

This study fully supports previous recommendations made by Odo et al. 2021 [50] to improve the DHS questionnaire, to add further questions regarding cooking and heating practices (e.g., stove and multiple fuel stacking) to allow for a more comprehensive assessment; which was an inherent limitation within this study. In addition, we highlight the need to document the prevailing weather/season at time of interview within the DHS survey, as climatic conditions are known to influence ARI risk [23], cooking location [51,52] fuel type and dryness of wood, enabling greater insight into the current situation at the time of interview. Having an improved undertaking of the contemporary situation at the time of interview would reduce some of the uncertainties around the self-reported measures collected. Although collection of self-reported respiratory symptoms lack a definitive clinical diagnosis and biological specificity [53], uncertainty is reduced by obtaining maternally reported in the two week prior to the interview, allowing for diagnosis in a large proportion of surveyed children. In addition, ARI risk and proportion of cleaner energy source usage could have changed over time, with the included surveys being undertaken between 2010 and 2018; however, all surveys were undertaken with the same methodology. Despite the limitations described, the DHS data provides an opportunity to analysis data which has a large population-based samples from multiple settings, an excellent response rate and national coverage and the use of uniform surveys, with robust fieldworker training and coordination [50,54,55].

## 5. Conclusions

Replacing solid biomass cooking with cleaner energy source alternatives will likely reduce the incidence of ALRI and ARI in children under five years, in resource poor settings worldwide. Any further structural, fiscal and behavioural cleaner cooking energy source interventions should consider the country level cultural and situational factors associated sustained clean energy source uptake. Urban settings should be considered for prioritised targeting of fuel transition policies to deliver maximum child health benefits. Future mixed-methods research should consider implementation, adaptation and adverse or unintended events of cleaner cooking energy source transition, to have sustained uptake for maximum public health benefit.

## Figures and Tables

**Table 1:** Descriptive statistics for ARI and ALRI within the pooled dataset (N = 353,802).

	ARI N = 299,118			ALRI N = 298,841		
Explanatory Variables	Yes N = 32,438 (10.8%)	No N = 266,679 (89.2%)	*p* Value	Yes N = 19,426 (6.5%)	No N = 279,415 (93.5%)	*p* Value
**Cooking energy source**			0.012			0.081
*Clean*	6547 (20.2%)	47,187 (17.7%)		3770 (19.4%)	49,921 (17.9%)	
*Biomass*	25,824 (79.8%)	219,015 (82.3%)		15,615 (80.6%)	228,992 (82.1%)	
*Missing*	67	477		41	502	
**Child age (years)**			<0.001			<0.001
*<1*	7572 (23.3%)	55,614 (20.9%)		4441 (22.9%)	58,720 (21.0%)	
*1*	7873 (24.3%)	53,320 (20.0%)		5102 (26.3%)	56,039 (20.1%)	
*2*	6476 (20.0%)	51,828 (19.4%)		3913 (20.1%)	54,349 (19.5%)	
*3*	5682 (17.5%)	53,057 (19.9%)		3266 (16.8%)	55,397 (19.8%)	
*4*	4835 (14.9%)	52,860 (19.8%)		2703 (13.9%)	54,910 (19.7%)	
**Sex of child**			<0.001			<0.001
*Male*	16,811 (51.8%)	134,922 (50.6%)		10,099 (52.0%)	141,489 (50.6%)	
*Female*	15,627 (48.2%)	131,757 (49.4%)		9327 (48.0%)	137,926 (49.4%)	
**Mode of delivery**			<0.001			<0.001
*Vaginal*	28,437 (88.9%)	244,809 (93.0%)		17,000 (89.2%)	255,981 (92.8%)	
*Caesarean*	3557 (11.1%)	18,436 (7.0%)		2065 (10.8%)	19,917 (7.2%)	
*Missing*	444	3435		362	3517	
**Breastfeeding status**			<0.001			<0.001
*Ever breast fed*	25,602 (96.4%)	219,737 (96.3%)		15,390 (96.1%)	229,736 (96.3%)	
*Never breast fed*	953 (3.6%)	8461 (3.7%)		620 (3.9%)	8782 (3.7%)	
*Missing*	5883	38,481		3416	40,896	
**Maternal age (Years)**			<0.001			<0.001
*15–24*	10,545 (32.5%)	75,517 (28.3%)		6260 (32.2%)	79,685 (28.5%)	
*25–35*	16,650 (51.3%)	143,162 (53.7%)		10,014 (51.5%)	149,672 (53.6%)	
*36–49*	5244 (16.2%)	48,001 (18.0%)		3152 (16.2%)	50,057 (17.9%)	
**Maternal level of education**			<0.001			<0.001
*No education*	7686 (23.7%)	92,024 (34.5%)		4751 (24.5%)	94,882 (34.0%)	
*Primary*	13,026 (40.2%)	88,356 (33.1%)		7825 (40.3%)	93,467 (33.5%)	
*Secondary or Higher*	11,726 (36.1%)	86,272 (32.4%)		6851 (35.3%)	91,038 (32.6%)	
*Missing*	0	29		0	29	
**Household wealth index**			<0.001			<0.001
*Lowest*	7902 (24.4%)	60,168 (22.6%)		4977 (25.6%)	63,041 (22.6%)	
*Low*	7335 (22.6%)	57,061 (21.4%)		4446 (22.9%)	59,886 (21.4%)	
*Middle*	6744 (20.8%)	53,640 (20.1%)		4029 (20.7%)	56,295 (20.1%)	
*High*	5850 (18.0%)	50,981 (19.1%)		3391 (17.5%)	53,379 (19.1%)	
*Highest*	4607 (14.2%)	44,829 (16.8%)		2582 (13.3%)	46,814 (16.8%)	
**Place of residence**			<0.001			<0.001
*Urban*	11,387 (35.1%)	90,829 (34.1%)		6565 (33.8%)	95,551 (34.2%)	
*Rural*	21,051 (64.9%)	175,850 (65.9%)		12,861 (66.2%)	183,864 (65.8%)	
**Household smoking**						
*No*	18,370 (73.7%)	182,328 (74.0%)		10,514 (71.8%)	189,978 (74.1%)	
*Yes*	6561 (26.3%)	63,968 (26.0%)		4135 (28.2%)	66,340 (25.9%)	
*Missing*	7507	20,384		4777	23,098	
**Number of households members**			<0.001			<0.001
*≤6*	19,835 (61.2%)	155,110 (58.2%)		11,834 (60.9%)	162,905 (58.3%)	
*≥6*	12,592 (38.8%)	111,369 (41.8%)		7586 (39.1%)	116,305 (41.7%)	
*Missing*	11	201		6	205	
**Cooking location**			<0.001			<0.001
*Indoors*	24,520 (75.8%)	188,269 (70.8%)		14,657 (75.7%)	197,942 (71.0%)	
*Outdoors*	7826 (24.2%)	77,651 (29.2%)		4702 (24.3%)	80,690 (29.0%)	
*Missing*	92	759		67	783	
**Paternal level of education**			<0.001			<0.001
*No education*	5692 (20.4%)	71,767 (30.7%)		3505 (20.8%)	73,897 (30.2%)	
*Primary*	10,201 (36.6%)	69,809 (29.9%)		6226 (37.0%)	73,718 (30.1%)	
*Secondary or Higher*	11,946 (42.9%)	92,192 (39.4%)		7110 (42.2%)	96,957 (39.6%)	
*Missing*	4599	32,911		2585	34,844	

**Table 2:** Adjusted odds ratio (AOR) with 95% CI for the association of ARI and ALRI in children aged under five years with biomass cooking compared to cleaner cooking.

	ARI (N = 298,379)		ALRI (N = 298,126)	
	AOR (95%CI)	*p* Value	AOR (95%CI)	*p* Value
**Cooking energy source**				
*Clean*	*Ref.*		*Ref.*	
*Biomass*	**1.17 (1.09–1.25)**	**<0.001**	**1.16 (1.07–1.25)**	**<0.001**
**Child age (years)**				
*<1*	**0.95 (0.91–0.99)**	**0.024**	**0.86 (0.82–0.91)**	**<0.001**
*1*	*Ref.*		*Ref.*	
*2*	**0.85 (0.81–0.88)**	**<0.001**	**0.79 (0.75–0.84)**	**<0.001**
*3*	**0.74 (0.71–0.78)**	**<0.001**	**0.66 (0.62–0.70)**	**<0.001**
*4*	**0.64 (0.61–0.67)**	**<0.001**	**0.56 (0.52–0.59)**	**<0.001**
**Sex of child**				
*Female*	*Ref.*		*Ref.*	
*Male*	**1.05 (1.02–1.09)**	**0.001**	**1.06 (1.02–1.10)**	**0.002**
**Mode of delivery**				
*Vaginal*	*Ref.*		*Ref.*	
*Caesarean*	**1.07 (1.01–1.14)**	**0.02**	1.02 (0.94–1.09)	0.67
**Maternal age (Years)**				
*15–24*	*Ref.*		*Ref.*	
*25–35*	**0.95 (0.92–0.99)**	**0.011**	0.99 (0.95–1.04)	0.703
*36–49*	**0.93 (0.89–0.98)**	**0.009**	0.98 (0.92–1.05)	0.585
**Maternal level of education**				
*No education*	*Ref.*		*Ref.*	
*Primary*	**1.22 (1.15–1.28)**	**<0.001**	**1.18 (1.11–1.26)**	**<0.001**
*Secondary or Higher*	**1.13 (1.07–1.20)**	**<0.001**	1.06 (0.99–1.14)	0.103
**Household wealth index**				
*Lowest*	*Ref.*		*Ref.*	
*Low*	0.99 (0.94–1.04)	0.624	0.95 (0.90–1.01)	0.091
*Middle*	0.98 (0.93–1.04)	0.509	**0.93 (0.87–1.00)**	**0.04**
*High*	**0.92 (0.87–0.99)**	**0.018**	**0.86 (0.80–0.93)**	**<0.001**
*Highest*	**0.87 (0.80–0.94)**	**0.001**	**0.78 (0.71–0.86)**	**<0.001**
**Place of residence**				
*Rural*	*Ref.*		*Ref.*	
*Urban*	0.98 (0.93–1.03)	0.387	1.01 (0.95–1.07)	0.755
**Number of households members**				
*≤6*	*Ref.*		*Ref.*	
*≥6*	0.99 (0.95–1.02)	0.440	0.98 (0.94–1.02)	0.367
**Cooking location**				
*Indoors*	*Ref.*		*Ref.*	
*Outdoors*	0.97 (0.93–1.02)	0.223	0.99 (0.93–1.04)	0.629

Abbreviation: N = number of observations, AOR = Adjusted Odds Ratio, 95% CI = 95% confidence interval. *Ref.* = Reference group. The bold is for the results to illustrate significant results below *p* = 0.05. Unadjusted results: Appendix A: Table A1.

**Table 3:** Adjusted odds ratio (AOR) with 95% CI for the association of ARI and ALRI in children aged under five years with biomass cooking compared to cleaner cooking for rural and urban areas.

Analysis (N)	ARI		ALRI	
	AOR (95%CI)	*p* Value	AOR (95%CI)	*p* Value
**Sub-analysis**				
Urban areas only (*n* = 101,444)	**1.16 (1.06–1.28)**	**0.001**	**1.14 (1.02–1.27)**	**0.018**
Rural areas only (*n* = 196,682)	**1.14 (1.03–1.26)**	**0.010**	1.13 (1.00–1.27)	0.051

Abbreviation: N = number of observations, AOR = Adjusted Odds Ratio, 95% CI = 95% confidence interval. The bold is for the results to illustrate significant results below *p* = 0.05. *n* = Number of observations within the sub-analysis.

## Data Availability

Data is freely and publicly available from https://dhsprogram.com/data/ (accessed on 28 February 2021).

## Appendix A

**Table A1:** Unadjusted odds ratio (OR) with 95% CI for the association of ARI and ALRI in children aged under five years with biomass cooking compared to cleaner cooking.

	ARI		ALRI	
	OR (95%CI)	*p* Value	OR (95%CI)	*p* Value
**Cooking energy source (Kerosene categorised as biomass energy source)**				
*Clean*	*Ref.*		*Ref.*	
*Biomass*	**0.85 (0.80–0.90)**	**<0.001**	**0.90 (0.85–0.96)**	**0.001**
**Cooking energy source (Kerosene categorised as clean energy source)**				
*Clean*	*Ref.*		*Ref.*	
*Biomass*	**0.89 (0.84–0.94)**	**<0.001**	0.95 (0.89–1.00)	0.067
**Child age (years)**				
*<1*	**0.92 (0.88–0.96)**	**<0.001**	**0.83 (0.79–0.87)**	**<0.001**
*1*	*Ref.*		*Ref.*	
*2*	**0.85 (0.81–0.88)**	**<0.001**	**0.79 (0.75–0.83)**	**<0.001**
*3*	**0.73 (0.69–0.76)**	**<0.001**	**0.65 (0.61–0.68)**	**<0.001**
*4*	**0.62 (0.59–0.65)**	**<0.001**	**0.54 (0.51–0.57)**	**<0.001**
**Sex of child**				
*Female*	*Ref.*		*Ref.*	
*Male*	**1.05 (1.02–1.08)**	**0.001**	**1.06 (1.02–1.09)**	**0.003**
**Mode of delivery**				
*Vaginal*	*Ref.*		*Ref.*	
*Caesarean*	**1.66 (1.57–1.76)**	**<0.001**	**1.56 (1.46–1.67)**	**<0.001**
**Breastfeeding status**				
*Ever breast fed*	*Ref.*		*Ref.*	
*Never breast fed*	0.97 (0.87–1.07)	0.528	**1.56 (1.46–1.67)**	**<0.001**
**Maternal age (Years)**				
*15–24*	*Ref.*		*Ref.*	
*25–35*	**0.83 (0.80–0.86)**	**<0.001**	**1.67 (1.58–1.77)**	**<0.001**
*36–49*	**0.78 (0.75–0.82)**	**<0.001**	**1.50 (1.41–1.60)**	**<0.001**
**Maternal level of education**				
*No education*	*Ref.*		*Ref.*	
*Primary*	**1.77 (1.68–1.85)**	**<0.001**	**1.67 (1.58–1.77)**	**<0.001**
*Secondary or Higher*	**1.63 (1.54–1.72)**	**<0.001**	**1.50 (1.41–1.60)**	**<0.001**
**Household wealth index**				
*Lowest*	*Ref.*		*Ref.*	
*Low*	0.98 (0.93–1.03)	0.403	**0.94 (0.89–1.00)**	**0.044**
*Middle*	0.96 (0.90–1.01)	0.131	**0.91 (0.85–0.97)**	**0.003**
*High*	**0.87 (0.82–0.93)**	**<0.00**	**0.80 (0.75–0.86)**	**<0.001**
*Highest*	**0.78 (0.73–0.84)**	**<0.001**	**0.70 (0.65–0.75)**	**<0.001**
**Place of residence**				
*Rural*	*Ref.*		*Ref.*	
*Urban*	1.05 (1.00–1.10)	0.065	0.98 (0.93–1.04)	0.526
**Household smoking**				
*No*	*Ref.*		*Ref.*	
*Yes*	1.03 (0.99–1.08)	0.132	**1.15 (1.09–1.21)**	**<0.001**
**Number of households members**				
*≤6*	*Ref.*		*Ref.*	
*≥6*	**0.88 (0.85–0.92)**	**<0.001**	**0.90 (0.86–0.94)**	**<0.001**
**Cooking location**				
*Indoors*	*Ref.*		*Ref.*	
*Outdoors*	**0.77 (0.74–0.81)**	**<0.001**	**0.79 (0.75–0.83)**	**<0.001**
**Paternal level of education**				
*No education*	*Ref.*		*Ref.*	
*Primary*	**1.84 (1.74–1.95**	**<0.001**	**1.78 (1.67–1.90)**	**<0.001**
*Secondary or Higher*	**1.63 (1.54–1.73)**	**<0.001**	**1.55 (1.45–1.65)**	**<0.001**

Abbreviations: OR = Unadjusted Odds Ratio, 95% CI = 95% confidence interval. *Ref.* = Reference group. The bold is for the results to illustrate significant results below *p* = 0.05.

**Table A2:** Adjusted odds ratio (AOR) with 95% CI of the exploratory analyses for the association of ARI and ALRI in children aged under five years with biomass cooking compared to cleaner cooking.

Analysis (N)	ARI		ALRI	
	AOR (95%CI)	*p* Value	AOR (95%CI)	*p* Value
**Exploratory analyses**				
Controlling for breastfeeding ^a^ (*n* = 255,588)	**1.14 (1.07–1.23)**	**<0.001**	**1.16 (1.07–1.26)**	**0.001**
Controlling for household’s smoking ^b^ (*n* = 272,852)	**1.13 (1.06** **–** **1.22)**	**0.001**	**1.13 (1.04** **–** **1.24)**	**0.005**
Controlling for husband’s education ^c^ (*n* = 179,330)	**1.13 (1.06** **–** **1.22)**	**0.001**	**1.13 (1.04** **–** **1.23)**	**0.003**

Abbreviation: N = number of observations, AOR = Adjusted Odds Ratio, 95% CI = 95% confidence interval. (a) Excluded: Albania 2017–2018, Angola 2015–2016, Burkina Faso 2010, Mozambique 2011, Myanmar 2015–2016, Nepal 2016, Senegal 2017, Zambia 2013–2014, Papua New Guinea 2016–2017, Bangladesh 2014, Dominican Republic 2013 and Peru 2012; (b) Excluded: Guatemala 2014–2015, Philippines 2013, Kenya 2014, Bangladesh 2014, Dominican Republic 2013 and Peru 2012; (c) Excluded: Malawi 2015–2016, Kenya 2014 and Papua New Guinea 2016–2017. The bold is for the results to illustrate significant results below *p* = 0.05.

**Table A3:** Adjusted odds ratio (AOR) with 95% CI for the association of ARI and ALRI in children aged under five years with biomass cooking compared to cleaner cooking, where kerosene is classified as a cleaner energy source.

	ARI		ALRI	
	AOR (95%CI)	*p* Value	AOR (95%CI)	*p* Value
	N = 298,379		N = 298,126	
**Cooking energy source**				
*Clean*	*Ref.*		*Ref.*	
*Biomass*	**1.20 (1.12–1.27)**	**<0.001**	**1.19 (1.10–1.28)**	**<0.001**
**Child age (years)**				
*<1*	**0.95 (0.91–0.99)**	**0.024**	**0.86 (0.81–0.91)**	**<0.001**
*1*	*Ref.*		*Ref.*	
*2*	**0.84 (0.81–0.88)**	**<0.001**	**0.79 (0.75–0.84)**	**<0.001**
*3*	**0.74 (0.71–0.78)**	**<0.001**	**0.66 (0.62–0.70)**	**<0.001**
*4*	**0.64 (0.61–0.67)**	**<0.001**	**0.56 (0.52–0.59)**	**<0.001**
**Sex of child**				
*Female*	*Ref.*		*Ref.*	
*Male*	**0.74 (0.71–0.78)**	**<0.001**	**0.66 (0.62–0.70)**	**<0.001**
**Mode of delivery**				
*Vaginal*	*Ref.*		*Ref.*	
*Caesarean*	**1.08 (1.01–1.14)**	**0.018**	1.02 (0.95–1.09)	0.646
**Maternal age (Years)**				
*15*–*24*				
*25*–*35*	**0.95 (0.92–0.99)**	**0.011**	0.99 (0.95–1.04)	0.719
*36*–*49*	**0.93 (0.89–0.98)**	**0.009**	0.98 (0.92–1.05)	0.59
**Maternal level of education**				
*No education*	*Ref.*		*Ref.*	
*Primary*	**1.22 (1.16–1.29)**	**<0.001**	**1.18 (1.11–1.26)**	**<0.001**
*Secondary or Higher*	**1.14 (1.07–1.21)**	**<0.001**	1.07 (0.99–1.15)	0.088
**Household wealth index**				
*Lowest*	*Ref.*		*Ref.*	
*Low*	0.99 (0.94–1.04)	0.657	0.95 (0.90–1.01)	0.101
*Middle*	0.98 (0.93–1.04)	0.577	**0.94 (0.88–1.00)**	**0.05**
*High*	**0.93 (0.87–0.99)**	**0.031**	**0.87 (0.80–0.94)**	**<0.001**
*Highest*	**0.88 (0.81–0.95)**	**0.001**	**0.79 (0.72–0.87)**	**<0.001**
**Place of residence**				
*Rural*	*Ref.*		*Ref.*	
*Urban*	0.99 (0.94–1.04)	0.617	1.02 (0.96–1.08)	0.537
**Number of households members**				
*≤6*				
*≥6*	0.98 (0.95–1.02)	0.341	0.98 (0.93–1.02)	0.295
**Cooking location**				
*Indoors*	*Ref.*		*Ref.*	
*Outdoors*	0.97 (0.93–1.01)	0.165	0.98 (0.93–1.04)	0.528
**Sub-analysis ***				
**Urban areas only (*n* = 101,444)**	**1.18 (1.08–1.29)**	**<0.001**	**1.17 (1.05–1.30)**	**0.003**
**Rural areas only (*n* = 196,682)**	**1.17 (1.06–1.29)**	**0.002**	**1.15 (1.02–1.29)**	**0.024**

Abbreviation: N = number of observations, AOR = Adjusted Odds ratio, 95% CI = 95% confidence interval. *Ref.* = Reference group. * Results for Sub-analysis are biomass cooking compared to cleaner cooking. *n* = Number of observations within the sub-analysis. The bold is for the results to illustrate significant results below *p* = 0.05.
